# Teachers at vocational schools as ambassadors for physical activity? Developing an empowering webinar

**DOI:** 10.1093/eurpub/ckac131.321

**Published:** 2022-10-25

**Authors:** V Kaiser, E Foitzik, H Hassel

**Affiliations:** Institute for Applied Health Sciences, Coburg University of Applied Sciences and Art, Coburg, Germany

## Abstract

**Background:**

Childcare centres have the potential to promote health-enhancing physical activity (PA) in children. However, institutions with need for action seem to be hard to reach. During the project “QueB 2 - developing quality with and through physical activity” different strategies to empower multipliers in spreading awareness for PA were tested. Currently, the aim of the project is to involve teachers at vocational schools for early childhood education in order to sensitize prospective educators and ensure quality in planning interventions.

**Methods:**

The webinar was developed in three steps: (1) Four workshops were implemented in two vocational schools by participating 80 future educators to train them in planning PA interventions. (2) The collected students’ recommendations and sample projects were categorized in eight steps of a theoretical model for project planning. (3) After finalizing the webinar, questionnaires were used to reflect on its application, utilization, and design.

**Results:**

The results of the second step were summarized in a manual for planning PA interventions in childcare centres. This forms the basis of the webinar to enable teachers to disseminate PA promotion in class. The developed modules were installed at a website and provided for free in order to be used by the interested public. In step three, teachers tested the webinar and filled in the questionnaire. The majority perceived the webinar as useful, appreciated its clear structure and appealing design, and expressed an increased awareness of factors concerning PA in childcare centres.

**Conclusions:**

Teachers at vocational schools perceived the webinar as useful to integrate parts of planning PA interventions in childcare settings in the curriculum. Transferring developed manuals and training modules to multipliers by providing an appealing, informative webinar seem to be a successful and sustainable way to ensure quality in planning processes.

**Key messages:**

• Involving teachers at vocational schools as multipliers for PA in childcare centres by performing a webinar is a sustainable strategy to maintain quality in intervention planning.

• Early childhood education students can act as ambassadors for PA.

